# Up‐regulated Cx43 phosphorylation at Ser368 prolongs QRS duration in myocarditis

**DOI:** 10.1111/jcmm.13631

**Published:** 2018-04-17

**Authors:** Chunlian Zhong, He Chang, Yang Wu, Li Zhou, Yan Wang, Mingyan Wang, Peng Wu, Zhi Qi, Jun Zou

**Affiliations:** ^1^ Department of Basic Medical Sciences Medical College of Xiamen University Xiamen China; ^2^ Department of Cardiology Affiliated Cardiovascular Hospital of Xiamen University Xiamen China

**Keywords:** connexin 43, experimental autoimmune myocarditis, IL‐1β, p38 MAPK, QRS duration

## Abstract

Prolongation of QRS duration in electrocardiogram is one of the risk factors for morbidity and mortality in many kinds of cardiac diseases. However, its molecular mechanism is unknown. In this study, utilizing experimental autoimmune myocarditis (EAM) as a disease model, we show that the prolongation of QRS duration is accompanied by elevated phosphorylation of connexin 43 (Cx43) at Ser368 (p^S^ ^368^Cx43). In cultured cells, inflammatory cytokine IL‐1β activates p38 MAPK to up‐regulate p^S^ ^368^Cx43 and impairs cell‐to‐cell communication. In isolated hearts of normal rats, perfusion of IL‐1β not only increases p^S^ ^368^Cx43 but also impairs cell‐to‐cell communication and prolongs QRS duration. Furthermore, blockade of p38 MAPK down‐regulates p^S^ ^368^Cx43, improves cell‐to‐cell communication and reduces QRS duration in EAM. These findings suggest that up‐regulation of p^S^ ^368^Cx43 by IL‐1β via p38 MAPK contributes to the prolongation of QRS duration and could be a therapeutic target for myocarditis‐induced prolongation of QRS duration.

## 1 INTRODUCTION

Myocarditis accounts for a large proportion of sudden cardiac deaths in young people without prior structural heart diseases. It has been reported that there is a higher incidence of arrhythmias or abnormal electrocardiogram (ECG) in the early stage of acute myocarditis.^1^ In patients with acute myocarditis, AV block, abnormal QRS complex, repolarization abnormality and ST‐segment elevation were the prevailing ECG features,^2^, ^3^ implying that multiple factors are involved in the myocarditis‐induced arrhythmias. Apparently, identifying each molecule target that is responsible for the corresponding component of the ECG abnormality in myocarditis is essential for understanding the underlying molecular mechanism.

The QRS complex is produced by waves of depolarization traversing the ventricular syncytium. As the most striking waveform within the ECG, the QRS complex reflects the electrical activity within the heart during the ventricular contraction. The time of its occurrence, which represents the time taken for the ventricular depolarization and propagation of the cardiac impulse throughout the ventricle,^4^ as well as its shape provides much information about the current state of the heart. Thus, the duration, amplitude and morphology of the QRS complex are useful in diagnosing cardiac arrhythmias. Even though the QRS complex is the most distinguishable component in the ECG, its clinical meanings have only been recognized gradually in recent two decades. It has been indicated in the 1990s that the QRS duration is significantly longer in patients with ventricular tachycardia.^5^ After that, the prolongation of QRS duration has been demonstrated in many kinds of cardiac diseases, such as coronary artery disease,^6^, ^7^ ischemic cardiomyopathy,^8^ myocardial infarction 9 and heart failure.^10^ Furthermore, the prolongation of QRS duration has been shown to be associated with death risk in right bundle branch block,^11^ worsen left ventricular function,^12^ atrial fibrillation,^13^ ventricular tachyarrhythmias,^14^ etc. For example, comparing the groups with duration of QRS ≥ 120 ms vs QRS < 120 ms (median follow‐up, 45 months), the mortality in patients with heart failure is 51% vs 34% and the sudden death rate is 25% vs 17% respectively.^8^ The relative risk of recurrent ventricular arrhythmia is nearly fourfold higher in patients who had the prolongation of QRS duration (≥120 ms) than in those with a normal QRS duration.^15^ A prolonged QRS duration in patients with heart failure has been shown to be associated with more advanced myocardial disease, worse left ventricular function, poorer prognosis and a higher all‐cause mortality rate compared with patients with a narrow QRS complex.^12^ The risk of inducible sustained monomorphic ventricular tachycardia increases by 2.4% for each 1 ms prolongation in QRS duration.^16^ For every 10 ms prolongation in QRS duration, mortality rate increases 10% for ventricular arrhythmias,^17^ 18%‐26% for bundle branch block^11^, ^18^ and 6% for myocardial infarction^9^ respectively. Thus, understanding the molecular mechanism of the prolongation of QRS duration is of clinical significance.

The duration of the QRS complex is determined by the ventricular depolarization and the propagation of the excitatory cardiac impulse throughout the ventricle. The prolongation of the QRS complex reflects ventricular conduction delay, a substrate for arrhythmogenicity.^19^ Gap junction channels form an intercellular pathway for electrical cell‐to‐cell coupling and are essential for normal cardiac impulse propagation. It has been shown that alterations in electrical coupling via gap junction channels contribute to abnormal conduction and arrhythmogenesis in the heart.^20^ Pathological alterations in connexin abundance or function can lead to slowing of conduction.^21^, ^22^ In mammalian ventricular muscle, connexin 43 (Cx43) is the predominant gap junction channel.^20^, ^23^ Impaired propagation, reflected in the prolongation of QRS duration, reduces coordinated ventricular contraction and forms a substrate for cardiac arrhythmias,^22^ which has been observed in cardiac‐restricted Cx43 “knockout” mice.^21^ Homozygous ablation of Cx43 in cardiomyocytes leads to low voltage QRS and significant prolongation of QRS duration.^24^ QRS duration was significantly prolonged in Cx43(+/−) mice than in wild type, but P‐wave duration and amplitude did not differ.^25^ Genetic knockout of Cx43 in mice is associated with conduction slowing, prolongation of QRS duration and increased susceptibility to ventricular arrhythmias.^21^, ^25^, ^26^ Replacement of Cx43 by Cx31 in the heart leads to significant prolongation of QRS duration.^27^ In addition, some preclinical and marketed drugs have been shown to cause QRS prolongation via Cx43 uncoupling.^28^ Therefore, as a principal conductor of intercellular current in the ventricle,^25^ Cx43 is one of the molecular determinants for the prolongation of QRS duration.

A model of rat experimental autoimmune myocarditis (EAM) resembles human giant cell myocarditis, and the recurrent form of EAM leads to dilated cardiomyopathy. In this study, utilizing EAM as a disease model, we show that the elevation of p^S368^Cx43 by IL‐1β via the p38 MAPK signalling pathway contributes to the prolongation of QRS duration in myocarditis.

## 2 MATERIALS AND METHODS

### 2.1 Induction of EAM model

Male Lewis rats (180‐200 g), aged 6‐8 weeks, purchased from Beijing Vital River Laboratory Animal Technology (Beijing, China) were maintained in Xiamen University animal experimental centre. All animal care and experiments were performed in accordance with procedures approved by the Animal Care and Use Committee of Xiamen University. Porcine cardiac myosin was prepared from the ventricular muscle of porcine hearts as previously described.^29^ To produce EAM, each rat was immunized on day 0 with 0.2 mL emulsion containing 1 mg porcine cardiac myosin with an equal volume of complete Freund's adjuvant supplemented with mycobacterium tuberculosis H37RA at a concentration of 10 mg/mL by a single subcutaneous injection in both footpads. The rats in the control group were only immunized with complete Freund's adjuvant.

### 2.2 In vivo administration of SB203580 to EAM rats

SB203580 was dissolved in DMSO, then intraperitoneally injected every 2 days at a concentration of 20 mg/kg to the rats from day 14 to day 20. The rats in the control group were intraperitoneally injected with same volume of DMSO. Relative mRNA expression and protein levels were measured by quantitative real‐time RT‐PCR and Western blot analysis.

### 2.3 Electrocardiography (ECG) recording

The ECG of all rats was recorded under light isoflurane anaesthesia and analysed off‐line using BL‐420S bio‐experiment system (Chengdu Techman Software Co. LTD, China). Heart rate (HR), P wave, QRS complex and PR interval were evaluated. QRS duration was measured in lead II from the earliest to the latest deflection of the QRS complex, while its amplitude was measured from the nadir to the top of each QRS complex.

### 2.4 Immunohistochemical analysis

Hearts were fixed in 10% formalin, embedded and cut into 5 μm thick sections. After deparaffinized and rehydrated, endogenous peroxidase was blocked by incubation in 3% H_2_0_2_ in methanol for 10 minutes. For antigen retrieval, the sections were heated at 120°C in citric acid buffer for 15 minutes and then cooled for 30 minutes at room temperature. After washed 3 times in PBS, the sections were blocked by 10% bovine serum albumin for 30 minutes and incubated with anti‐connexin43 antibody (Sigma, 1:2000), anti‐phospho‐Cx43 antibody (Ser368) (Cell Signalling, 1:100) overnight at 4°C. Afterwards, the sections were washed 3 times in PBS, followed by incubation with horseradish peroxidase‐conjugated secondary antibodies (Jackson, 1: 500) for 1 hour at room temperature. The immune reaction was performed using a DAB Kit (MXB, China) under microscope. The sections were counterstained with hematoxylin, gradient alcohol dehydration and mounted.

### 2.5 Western blot analysis

The cardiac ventricles of the rat and the cultured H9c2 cells (Chinese Academy of Sciences Cell Bank, Shanghai) were homogenized on ice and lysed in RIPA buffer (P0013B Beyotime, China) supplemented with proteinase and phosphatase inhibitors. Each protein in the sample was separated by 10% SDS‐PAGE gels and transferred to the PVDF membrane (Millipore, USA). The membranes were blocked by 5% skim milk in PBS containing 0.05% Tween 20 for 1 hour and incubated with anti‐GAPDH antibody (Abcam, 1:5000), anti‐connexin43 (Sigma, 1:10000), anti‐phospho‐Cx43 (Ser368) (Cell Signalling, 1:1000), anti‐p38 (Cell Signalling, 1:1000) and anti‐phospho‐p38 (Thr180/Tyr182) (Cell Signalling, 1:1000) overnight at 4°C. Afterwards, the membranes were washed in PBS containing 0.05% Tween 20, followed by incubation with horseradish peroxidase‐conjugated secondary antibodies (Jackson, 1:10000) for 1 hour at room temperature. The immunoreactivity was visualized by an enhanced chemiluminescence (ECL) advanced kit (Millipore). Signal intensities were analysed using Image J software.

### 2.6 Cell culture

H9c2 cells were cultured in Dulbecco's modified eagle's medium (DMEM) supplemented with 10% foetal bovine serum (FBS), 100 units/mL penicillin G and 100 mg/mL streptomycin at 37°C in a humidified atmosphere of 5% CO_2_. The medium was changed every 2‐3 days and the cells were subcultured regularly.

### 2.7 Fluorescent dye transfer assay of cell‐to‐cell communication

H9c2 cells were bathed in Hanks’ balanced salts solution (HBSS, Gibco, in mmol/L): 1.3 CaCl_2_, 0.8 MgSO_4_, 5.4 KCl, 0.4 KH_2_PO_4_, 136.9 NaCl, 0.3 Na_2_PO_4_, 10 D‐glucose and 4.2 NaHCO_3_. The pH and osmolarity of the bath and the pipette filling solution were adjusted to 7.4 and 295 mOsmol/L respectively. Microelectrodes (tip diameter, ~1 μm) were pulled from capillaries and backfilled with Lucifer Yellow (0.5%) dissolved in the pipette filling solution (in mmol/L): 100 K‐gluconate, 40 KCl, 5 Na_2_ATP, 2.5 MgCl_2_, 0.25 CaCl_2_, 1 BAPTA, 0.2 cGMP, 1 glucose and 10 HEPES. Whole‐cell patch‐clamp configuration was established on one of the H9c2 cells within the monolayer on a cover slip, where the cells were grown to over 90% confluence. Pipette filling solution containing 0.5% Lucifer Yellow was allowed to directly diffuse into the cell under whole‐cell configuration (EPC‐10) and through the gap junction channels into the adjacent cells. Ten minutes after establishment of the whole cell configuration, all Lucifer Yellow‐positive cells, irrespective of their absolute level of fluorescence, were counted using a fluorescence microscope to judge activity of the Cx43 gap junction channels as previously described.^30^

### 2.8 Preparation of isolated perfused heart

Rats were anesthetized with isoflurane after treatment with heparin (10 mg/kg) for 30 minutes. A thoracotomy was performed to excise the heart. During preparation, the heart was washed with cold Krebs‐Henseleit solution to decrease its contractility. Then, it was connected to Langendorff's apparatus and perfused with modified oxygenated Krebs‐Henseleit solution until the end of the experiment. The oxygenated Krebs‐Henseleit buffer contains (in mmol/L) 118 NaCl, 5.6 KCl, 2.2 CaCl_2_, 1.79 NaHCO_3_ and 12.6 glucose (pH 7.4). The pressure above the heart was maintained at a constant level of 1000 mm H_2_O and the temperature of the perfusion solution was maintained at 37.0 ± 0.5°C. The hearts were stabilized for 20 minutes, followed by perfusion with different solutions for 80 minutes. In control group, the isolated hearts were only perfused with modified Krebs‐Henseleit buffer. The surface ECG was continuously recorded from the heart through a multichannel physiological signal recording system (BL‐420S, Chengdu Techman Software Co.LTD, China), with one electrode positioned at the base and one at the apex of the heart.

### 2.9 Scrape‐loading dye transfer

The isolated heart was perfused for 10 minutes to rinse the blood and immersed in cold Krebs‐Henseleit solution for 10 minutes at 4°C to decrease its contractility. Then, the apex of heart was sectioned and incubated for 1 minutes in Lucifer yellow at 4°C according to a previously described method.^31^ Afterwards, the heart was immediately fixed in liquid nitrogen and embedded in OCT compound. The heart was sliced (50 μm) and imaged with fluorescence microscope.

### 2.10 Immunofluorescence analysis

Cryostat sections (5 μm) of hearts were fixed in 4% paraformaldehyde for 10 minutes, and processed for immunostaining as previously described.^32^ The H9c2 cells cultured on six‐well glass chamber slides were fixed in 4% paraformaldehyde, blocked by 10% bovine serum albumin for 30 minutes and stained with antibodies overnight at 4°C. Afterwards, the sections were washed 3 times in PBS and incubated with Alexa 549‐conjugated goat anti‐rabbit antibodies (Rockland) for 1 hour at room temperature. Nuclei were stained with DAPI. Slices were mounted with antifade mounting medium (Applygen, China) and analysed using Olympus microscope (IX51).

### 2.11 Statistical analysis

All data are shown as mean ± SEM. Data were analysed statistically by one‐way analysis of variance (ANOVA) followed by the Bonferroni test for multiple comparisons. Origin7.0 (Microcal Software, Inc.) or Prism 5.0 (GraphPad Software, Inc) software was used for statistical analyses. Values of *P *<* *.05 were considered to be statistically significant.

## 3 RESULTS

### 3.1 Experimental autoimmune myocarditis induces prolongation of QRS duration in ECG

**Figure 1:**
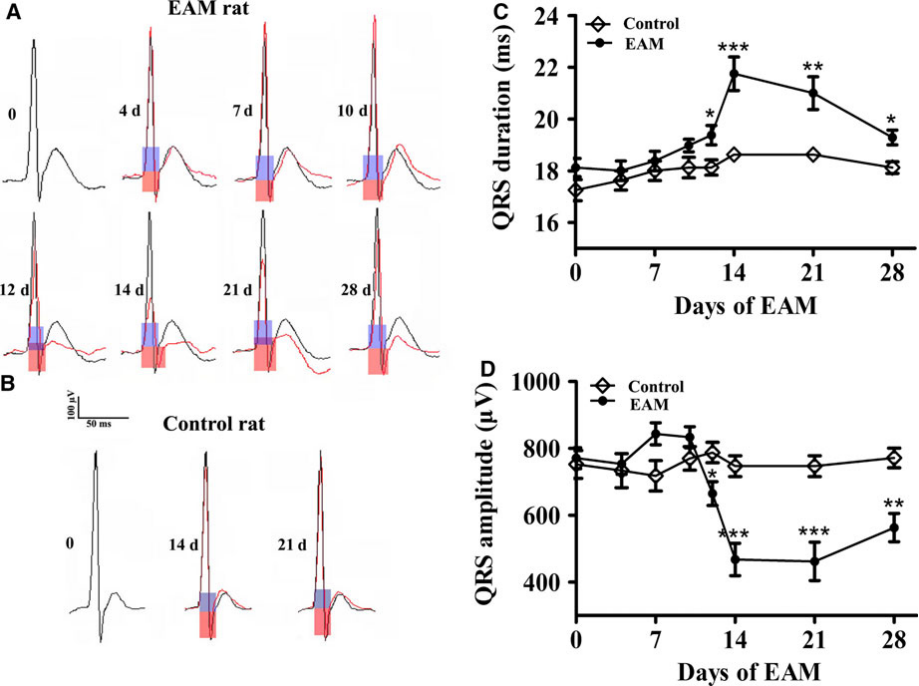
Experimental autoimmune myocarditis (EAM) induces prolongation of QRS duration in electrocardiogram (ECG). A and B, Representative QRS complex recorded from the same rat during the progression of EAM from day 0, 4, 7, 10, 12, 14, 21 and 28 (A) or a control rat on the corresponding day 0, 14 and 21 (B). Light blue box: QRS duration for day 0 of EAM or corresponding control rat; Light red box:QRS duration for EAM rat on day 4, 7, 10, 12, 14, 21 and 28 or corresponding control rat on day 14 and 21. C and D, QRS duration (C) and QRS amplitude (D) for the control and the EAM rats (n = 6‐8). *P < .05 vs day 0; **P <* *.01 vs day 0; ***P < .001 vs day 0

To investigate whether EAM could result in the prolongation of QRS duration, we carried out ECG recordings on the rats. Representative lead II ECG recordings on day 0 and during the progression of EAM is shown in Figure 1A. On day 0, the QRS duration was 18.1 ± 0.4 ms, which was almost not changed till day 10. From day 10 to 14 the QRS duration was prolonged from 19.0 ± 0.2 ms to the maximum on day 14 (21.8 ± 0.6 ms), then reduced to 21.0 ± 0.6 ms on day 21 and 19.3 ± 0.3 ms on day 28 (Figure 1C). QRS amplitude was 770 ± 31 μV on day 0, began to decrease from day 7 and reached minima on day 14 (467 ± 48 μV) (Figure 1D), indicating the appearance of low voltage QRS, which is usually along with that of the prolongation of QRS duration.^21^, ^24^ In contrast, the QRS duration and its amplitude were not changed on the corresponding day of the control rats (Figure 1B). Similar result has been reported in DBA/2 mice with experimentally induced myocarditis.^2^

### 3.2 Experimental autoimmune myocarditis induces up‐regulation of p^S368^Cx43

**Figure 2:**
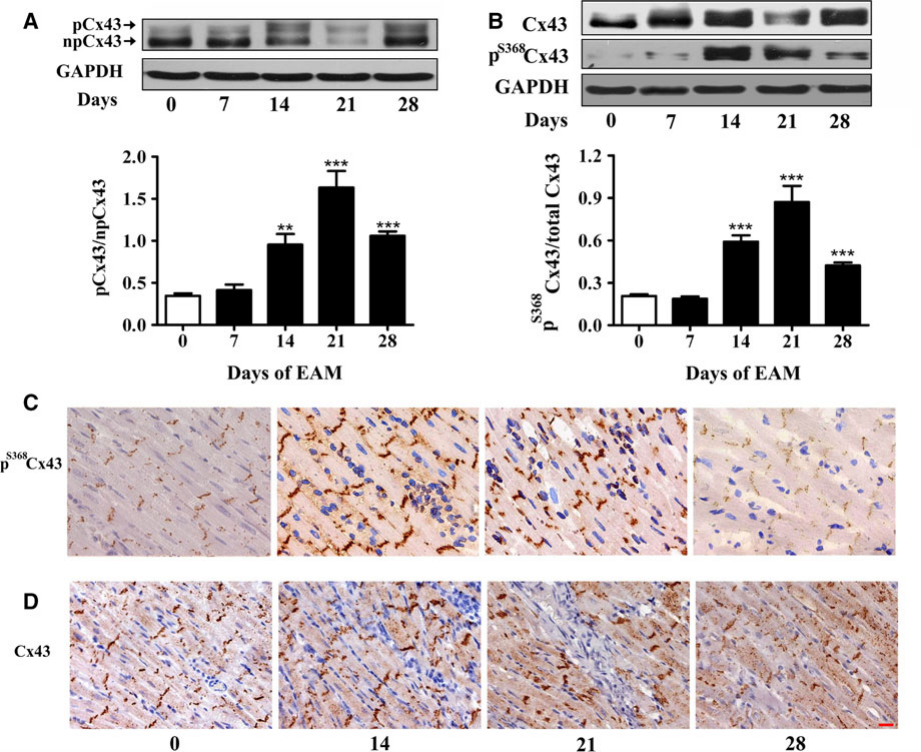
Experimental autoimmune myocarditis (EAM) induces up‐regulation of p^S^ ^368^Cx43. A, The ratio between phosphorylated and non‐phosphorylated form of Cx43 in the cardiac ventricles on day 0, 14, 21 and 28 of EAM. Upper panel: Western blot; Lower panel: Statistical summary. B, Relative p^S^ ^368^Cx43 level in the cardiac ventricles on day 0, 14, 21 and 28 of EAM (n = 6‐8). Upper panel: Western blot; Lower panel: Statistical summary. *P <* *.05 vs day 0; **P <* *.01 vs day 0; ***P <* *.001 vs day 0. C and D, Sections of stained p^S^ ^368^Cx43 (C, brown) and Cx43 (D, brown) in the longitudinal sections of the inflammatory area of cardiac ventricles on day 0, 14, 21 and 28 of EAM. Bar = 20 μm

Next, we investigated whether EAM affected Cx43, since it has been reported that the prolongation of QRS duration is closely associated with the dysfunction of Cx43.^21^, ^24^ Figure 2A showed that the ratio between phosphorylated and non‐phosphorylated Cx43 in the cardiac ventricles was significantly increased on day 14 and peaked on day 21, which was reduced on day 28 but was still higher than that of day 0. The relative level of the p^S368^Cx43 was significantly increased from 0.21 ± 0.01 on day 0 to 0.59 ± 0.05 on day 14, 0.87 ± 0.11 on day 21 and reduced to 0.42 ± 0.02 on day 28 (Figure 2B). In agreement with this result, immunohistochemical staining of the p^S368^Cx43 showed that the p^S368^Cx43 was obviously increased on day 14 and 21 of EAM (coloured brown in Figure 2C). As we have previously shown that the p^S368^Cx43 affects the distribution of Cx43 in H9c2 cells,^30^ we investigated the distribution of Cx43 in EAM. On day 0 of EAM, Cx43 situated predominantly as dense streaks at the intercalated discs of the ventricular myocardium. With the progression of EAM, Cx43 became extensively dispersed at the intercalated discs on day 14 and 21 and partly restored to the dense pattern on day 28 (Figure 2D). Because increase in the p^S368^Cx43 impairs Cx43‐mediated cell‐to‐cell communication,^32^, ^33^ these results imply that the Cx43‐mediated cell‐to‐cell communication between cardiomyocytes may be impaired in the progression of EAM. It is worth to note that these time‐dependent changes in the p^S368^Cx43 and Cx43 distribution were roughly coincided with the time course of the prolongation of QRS duration, implying a correlation between p^S368^Cx43, Cx43 and prolongation of QRS duration.

### 3.3 Experimental autoimmune myocarditis serum up‐regulates p^S368^Cx43 and reduces cell‐to‐cell communication via p38 MAPK signalling pathway

**Figure 3:**
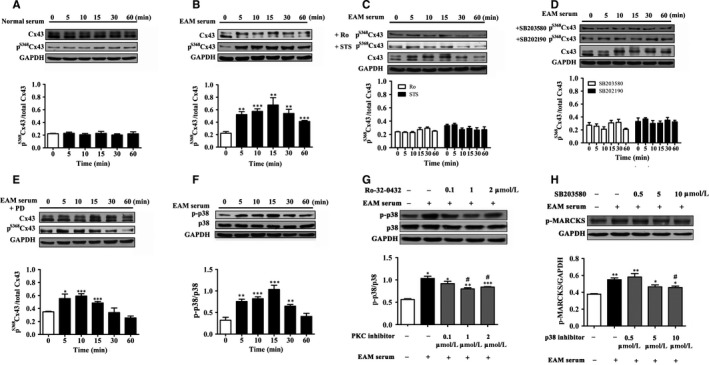
Experimental autoimmune myocarditis (EAM) serum up‐regulates p^S^ ^368^Cx43 through PKC/p38 signalling. A‐E, Time course of 0.25% serum from control group (A) as well as 0.25% EAM serum in the absence (B) and presence of PKC inhibitors (2 μmol/L Ro‐32‐0432 and 100 nmol/L STS), p38 inhibitors (10 μmol/L SB203580 and SB202190) (D) or Erk inhibitor (20 μmol/L PD98059) (E) on relative p^S^ ^368^Cx43 (n = 3‐4). (F) Time course of the EAM serum on phosphorylation of p38 MAPK (p‐p38) (n = 4). (G) Effect of PKC inhibitor (Ro‐32‐0432) on 0.25% EAM (15 minutes) induced phosphorylation of p38 MAPK (p‐p38). Cells were pretreated (30 minutes) with Ro‐32‐0432 (0.1, 1 and 2 μmol/L) (n = 3). (H) Effect of p38 inhibitor (SB203580) on EAM serum (15 minutes) induced phosphorylation of MARCKS (p‐MARCKS). Cells were pretreated (30 minutes) with SB203580 (0.5, 5 and 10 μmol/L) (n = 3). Upper panel: Western blot; Lower panel: Statistical summary. *P <* *.05 vs control; **P <* *.01 vs control; ***P <* *.001 vs control; ^#^ *P *<* *.05 vs EAM serum

To find a clue of why EAM could induce up‐regulation of the p^S368^Cx43, we studied the effect of serum from rats at the most severe inflammatory stage of EAM (day 14) on the p^S368^Cx43 in cultured H9c2 cells, a standard cardiac cell line derived from embryonic cells. Treatment of the cells with the EAM serum induced a time‐dependent increase in the p^S368^Cx43 level (Figure 3B), which was not observed in the cells treated with the serum from the control rats (Figure 3A). The level of p^S368^Cx43 began to increase after 5 minutes of the treatment, peaked after 15 minutes and gradually decreased from then on (Figure 3B). Pre‐incubation of the cells with PKC inhibitors (Ro‐32‐0432 and STS) for 30 minutes blocked the EAM serum induced increase in the p^S368^Cx43 level (Figure 3C), suggesting that the PKC signalling pathway is involved in the regulation of p^S368^Cx43. As p38 MAPK plays an important role in inflammatory diseases, we also investigated whether the p38 MAPK is involved in the regulation of p^S368^Cx43. Pre‐treatment of the cells with p38 MAPK inhibitors (SB203580 and SB202190) abolished the effect of the EAM serum on the p^S368^Cx43 (Figure 3D). In support of the role of p38 MAPK in the EAM serum induced up‐regulation of the p^S368^Cx43, treatment of cells with the EAM serum increased phosphorylation of p38 MAPK itself (Figure 3F), which indicates the activation of the p38 MAPK. It should be noted that the time course of the EAM serum on the p^S368^Cx43 increase coincided with that of the p38 MAPK activation. In contrast, treatment with PD98059, a specific inhibitor of Erk (1/2), did not affect the effect of the EAM serum on the p^S368^Cx43 (Figure 3E), even though the EAM serum could phosphorylate Erk to make it activation (Figure S1). These results suggest that both PKC and p38 MAPK signalling pathways contribute to the elevation of the p^S368^Cx43 level induced by the myocarditis serum. In addition, we found that PKC inhibitor (Ro‐32‐0432) could partly suppress phosphorylation of p38 (p‐p38) induced by the EAM serum (Figure 3G). Similarly, p38 inhibitor (SB203580) could also partly suppress the EAM serum up‐regulated phosphorylation of MARCKS (p‐MARCKS, Figure 3H), which is a marker for PKC activation. These results imply that there might be interaction between p38 MAPK and PKC signalling pathways. As effect of PKC on the p^S368^Cx43 has been intensively studied,^30^, ^32^, ^33^ we focus on the effect of p38 MAPK on the p^S368^Cx43 in the following experiments.

**Figure 4:**
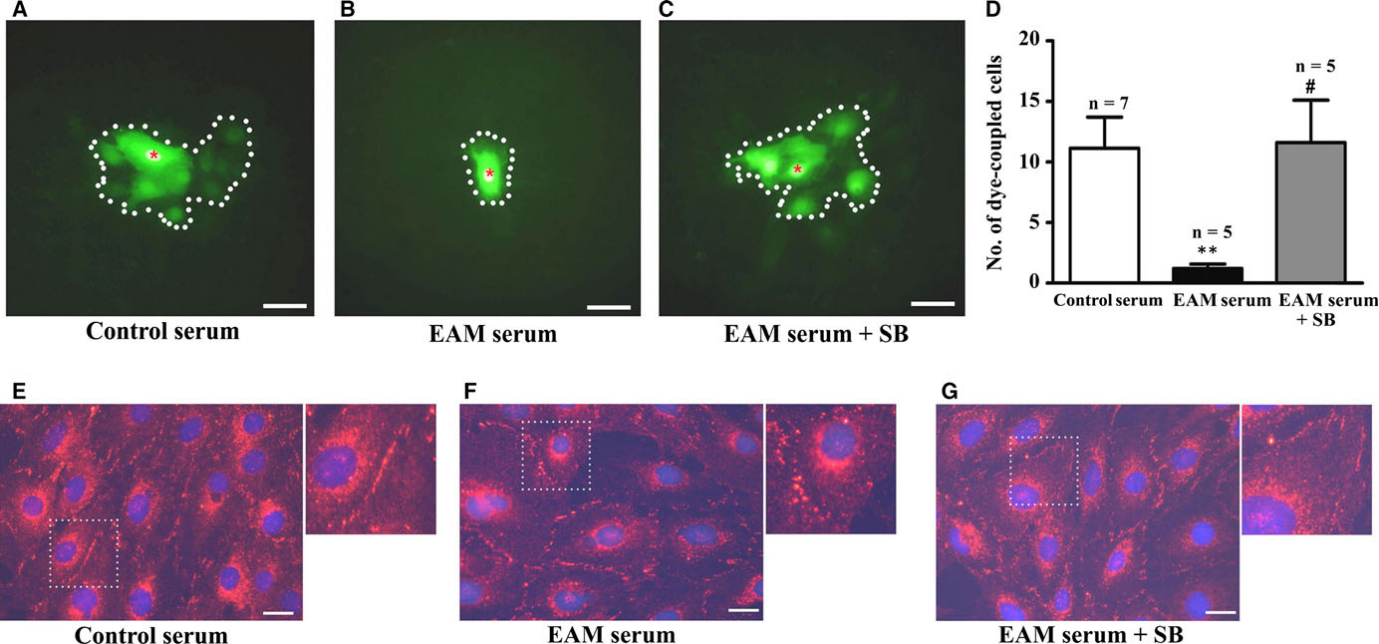
Experimental autoimmune myocarditis (EAM) serum suppresses cell‐to‐cell communication via p38 signalling. A‐C, Image of dye‐coupling between the cells treated with 0.25% serum from the control rats (A) and EAM (B) rats as well as 0.25% EAM serum in the presence of 10 μmol/L SB203580 (C). Dotted line: dye‐coupled cells. Red asterisk: dye‐injected cell. D, Statistical summary for dye coupled cells; *P <* *.01 vs control; ^#^ *P *<* *.05 vs EAM serum. E‐G, Immunofluorescent image of Cx43 in the cells treated with 0.25% serum from the control (E) and EAM (F) rats as well as 0.25% EAM serum in the presence of 10 μmol/L SB203580 (G). For greater clarity, areas in dotted squares are enlarged twofold in the upper right of each figure. Bar = 50 μm

Increase in the p^S368^Cx43 has been shown to decrease gap junction mediated cell‐to‐cell communication.^30^, ^32^, ^33^ To test whether the elevation of the p^S368^Cx43 level by the EAM serum could affect Cx43‐mediated cell‐to‐cell communication, we carried out fluorescent dye transfer assay on the H9c2 cells. Intracellular microinjection of Lucifer Yellow revealed that the dye diffused to 10.9 ± 2.3 cells treated with serum from control rats (Figure 4A and D) within 10 minutes. In the EAM serum treated cells, the dye spread into only 1.2 ± 0.6 cells (Figure 4B and D). Pre‐treatment of the cells with SB203580 increased the number of the dye‐coupled cells in the EAM serum treated group to 12.0 ± 3.7 (Figure 4C and D). Consistent with the dye transfer assay, immunofluorescent images showed that SB203580 recovered the distribution of Cx43 in the interfaces between the cells from the diffused manner in the EAM serum treated group (Figure 4F) to the condensed manner (Figure 4G), similar to that treated with the serum from the control rats (Figure 4E). These results suggest that the reduction of cell‐to‐cell communication by the EAM serum is due to its ability to elevate the p^S368^Cx43 level via the activation of p38 MAPK.

### 3.4 IL‐1β activates p38 MAPK, up‐regulates p^S368^Cx43 and reduces cell‐to‐cell communication in cultured cells

**Figure 5:**
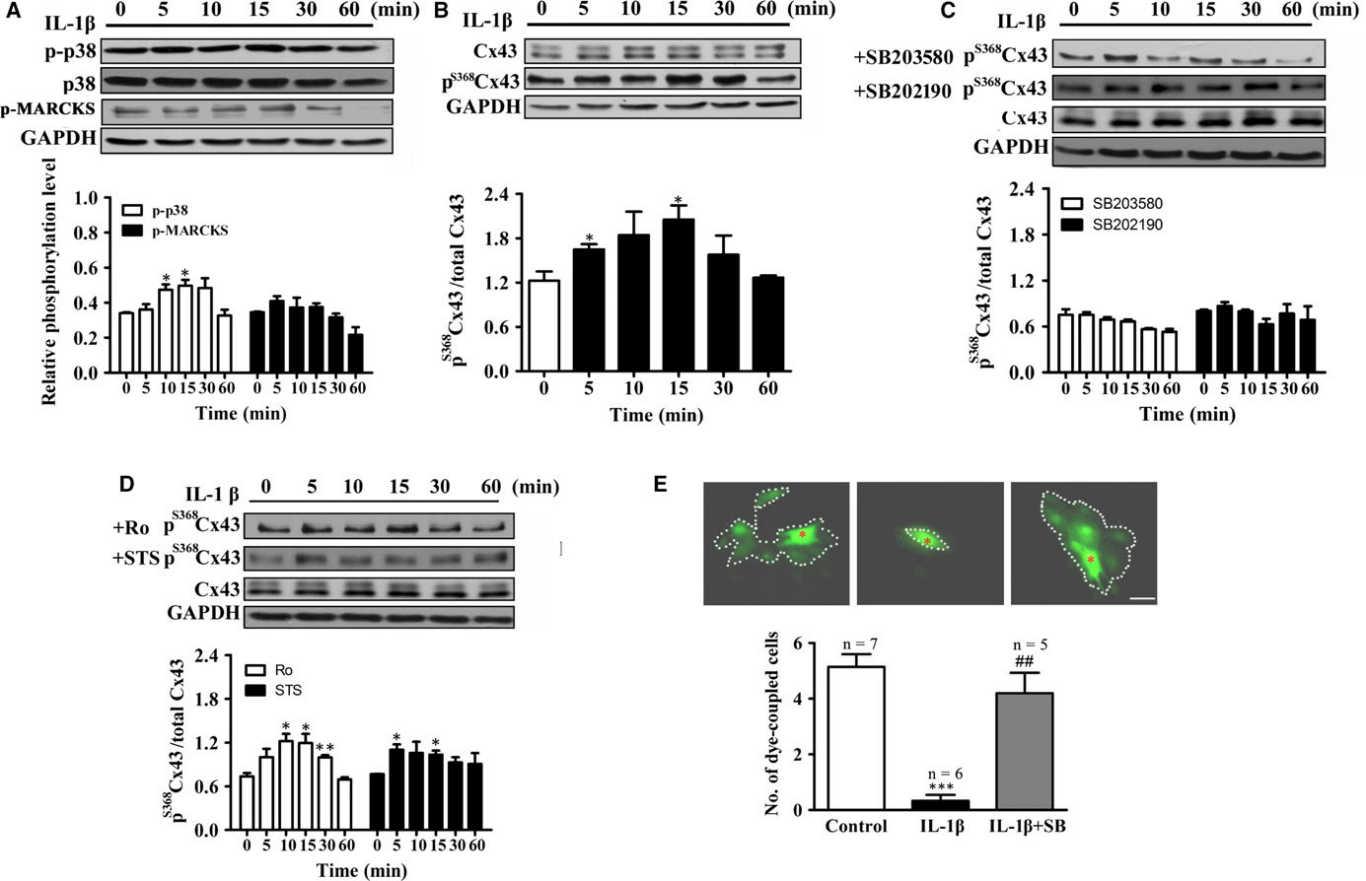
IL‐1β up‐regulates p^S^ ^368^Cx43 level and suppresses cell‐to‐cell communication via p38 MAPK. A, Time course of 10 ng/mL IL‐1β on p‐p38 (n = 3). Upper panel: Western blot; Lower panel: Statistical summary. B‐D, Time course of 10 ng/mL IL‐1β on relative p^S^ ^368^Cx43 (B), in the presence of p38 inhibitors (10 μmol/L SB203580 or SB202190) (n = 3) (C) or PKC inhibitors (2 μmol/L Ro‐32‐0432 or 100 nmol/L STS) (D). Upper panel: Western blot; Lower panel: Statistical summary. E, Effect of IL‐1β on the number of dye coupled cells in the absence or presence of 10 μmol/L SB203580. Dotted line: dye‐coupled cells. Red asterisk: dye‐injected cell. *P <* *.05 vs control; ***P <* *.001 vs control; ^##^ *P *<* *.01 vs IL‐1β. Bar = 50 μm

Cytokines, abundantly produced in the heart of myocarditis, play crucial roles in the pathophysiology of myocarditis. It has been reported that blocking either IL‐1β or TNF‐α related signalling pathways ameliorates the severity of myocarditis.^34^, ^35^, ^36^, ^37^ Besides, levels of inflammatory cytokines, such as TNF‐α and IL‐1β were raised in patients with acute myocarditis.^37^ We therefore examined whether cytokines in the EAM serum contribute to the EAM induced elevation of p^S368^Cx43. We found that treatment of the cells with IL‐1β increased the phosphorylated (p‐p38) and thus, activated form of p38 MAPK (Figure 5A). The activated p38 MAPK promoted the elevation of the p^S368^Cx43 with a maximal induction at 15 minutes (Figure 5B), the time course of which coincided with that of the EAM serum induced elevation of p^S368^Cx43 and p‐38 (Figure 3B and F). Furthermore, the effect of IL‐1β on the p^S368^Cx43 was inhibited by p38 inhibitors (SB203580 and SB202190) (Figure 5C), but not by PKC inhibitors (Ro‐32‐0432 and STS) (Figure 5D), suggested that the PKC pathway was not activated by IL‐1β. In addition, treatment of cell with IL‐1β reduced the number of dye‐coupled cells from 5.1 ± 0.5 of the control to 0.3 ± 0.2 (Figure 5E) within 10 minutes. Pre‐treatment of the cells with SB203580 increased the number of the dye‐coupled cells to 4.2 ± 0.7 (Figure 5E). In contrast, treatment of the cells with TNF‐α only slightly increased the p^S368^Cx43 and the phosphorylated form of p38 (Figure S2 A and C), which was inhibited by SB203580 as well (Figure S2 B). These results suggest that the effect of the EAM serum on the p^S368^Cx43 is mainly due to the ability of IL‐1β to activate p38 MAPK to phosphorylate Cx43 at Ser368. In agreement with this result, it has been reported in astrocytes that IL‐1β induces p38 activation and inhibits dye coupling of Cx43‐mediated gap junction.^38^

### 3.5 IL‐1β up‐regulates p^S368^Cx43, impairs cell‐to‐cell communication and prolongs QRS duration in isolated rat heart

**Figure 6:**
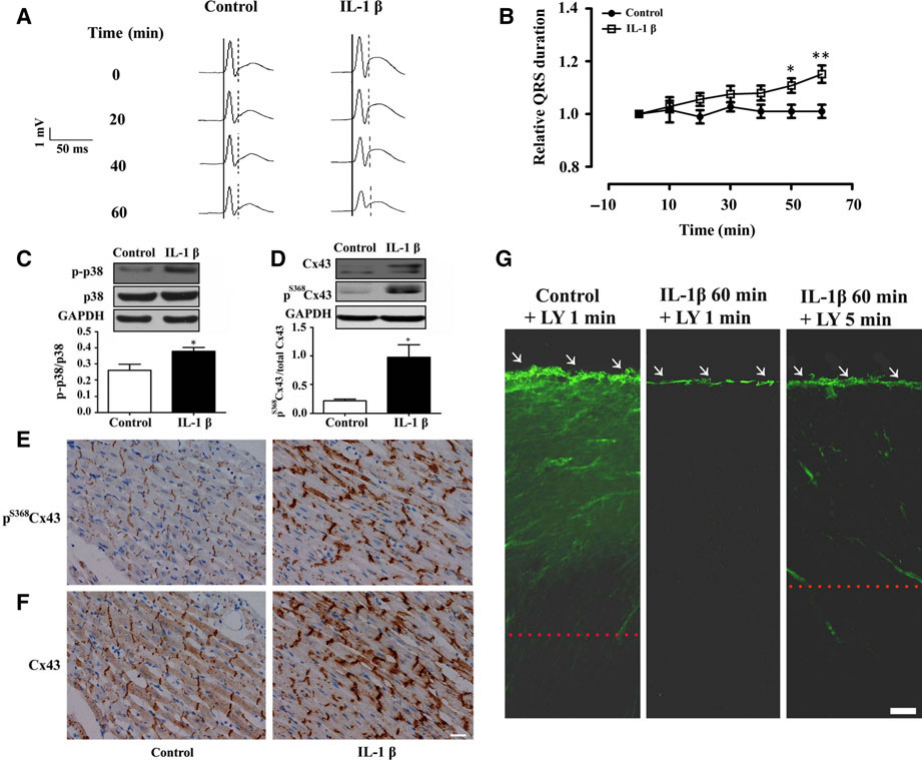
IL‐1β up‐regulates p^S^ ^368^Cx43 to cause prolongation of QRS duration in isolated normal heart. A and B, Representative electrocardiogram (ECG) recordings (A) and statistical summary (B, n = 6‐7) on the time course of QRS duration in isolated normal heart perfused with control solution or 10 ng/mL IL‐1β. The solid and doted lines in (A) indicate the period of QRS duration. *P < .05; **P <* *.01. C and D, Relative level of phosphorylation of p38 MAPK (p‐p38) (C, n = 6‐7) and p^S^ ^368^Cx43 (D, n = 6) in the cardiac ventricle of the isolated heart perfused with oxygenated Krebs‐Henseleit buffer or 10 ng/mL IL‐1β. Upper panel: Western blot; Lower panel: Statistical summary. E and F, Sections of stained p^S^ ^368^Cx43 (E, brown) and Cx43 (F, brown) in the longitudinal sections of cardiac ventricle in the isolated heart perfused with control solution or 10 ng/mL IL‐1β. Bar = 20 μm. G, Representative snapshots of dye (LY, Lucifer yellow) transfer for 1 or 5 minutes in the slice of normal heart, which was perfused with oxygenated Krebs‐Henseleit buffer (Control) or the buffer contained 10 ng/mL IL‐1β for 60 minutes. Bar = 100 μm. Dotted line: the edge of dye transferred. White arrows: cutting edge at the apex of the heart

In order to investigate whether the impairment of cell‐to‐cell communication at the cellular level could also apply to the heart level, we perfused isolated heart of normal rats with IL‐1β. Representative ECG recordings (Figure 6A) and data of relative QRS duration (Figure 6B) demonstrated that perfusion with IL‐1β gradually caused significant prolongation of QRS duration after 50 minutes, while perfused with control solution had no significant effect. Perfused with IL‐1β also caused significant activation of p38 MAPK (Figure 6C) and significant increase in the relative level of the p^S368^Cx43 in the isolated heart (Figure 6D). In addition, immunohistochemical staining of p^S368^Cx43 (Figure 6E) and Cx43 (Figure 6F) showed that IL‐1β caused distribution of both p^S368^Cx43 and Cx43 from a dense manner to a disperse manner at the intercalated discs of the ventricular myocardium. Furthermore, IL‐1β reduced diffusion of the fluorescent dye (Lucifer yellow) from the cut edge into the centre of the isolated heart within 1 minutes (middle panel, Figure 6G). In contrast, the dye diffused toward the heart centre deeply within 1 minutes in the control condition (left panel, Figure 6G). It is worthy to note that the dye could diffuse deeply if it was allowed to diffuse 5 minutes (right panel, Figure 6G), suggesting that the function of Cx43 is maintained somehow. These results strongly suggest that elevation of p^S368^Cx43 by IL‐1β directly impairs cell‐to‐cell communication and thus prolongs the QRS duration.

### 3.6 Blockade of p38 MAPK suppresses p^S368^Cx43, improves cell‐to‐cell communication and reduces QRS duration in EAM

**Figure 7:**
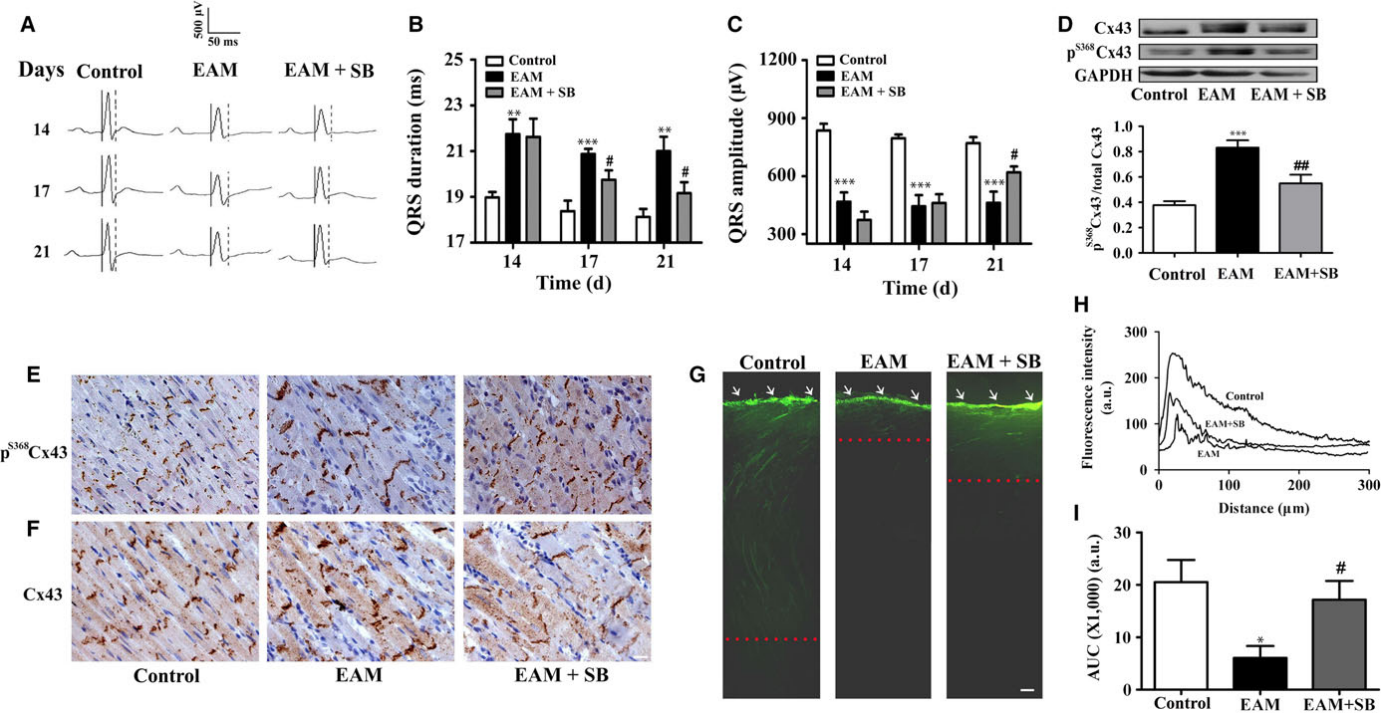
Blockade of p38 MAPK prevents the prolongation of QRS duration by suppressing the elevation of p^S^ ^368^Cx43 in EAM. A, Representative electrocardiogram (ECG) of control, EAM and SB203580 (20 mg/kg) treated EAM rats. SB203580 was intraperitoneally injected every two days to the rats from day 14 to day 20. Each set of ECG was recorded from the same rat on day 14, 17 and 21. The solid and doted lines indicate the period of QRS duration. B and C, Statistical summary on QRS duration (B) and QRS amplitude (C) from control, EAM and SB203580‐treated EAM rats (n = 6‐8). D, Western blot analysis of relative p^S^ ^368^Cx43 in the cardiac ventricles of control, EAM and SB203580‐treated EAM rats (n = 6‐8). Upper panel: Western blot; Lower panel: Statistical summary. **P <* *.01 vs control; ***P <* *.001 vs control; ^#^ *P *<* *.05 vs EAM; ^##^ *P *<* *.01 vs EAM. E and F, Immunohistochemical staining of p^S^ ^368^Cx43 (E, brown) and Cx43 (F, brown) in the ventricular sections of the control, EAM and SB203580‐treated EAM rats on day 21 of EAM (n = 6). Bar = 20 μm. G, Representative snapshots of dye (LY, Lucifer yellow) diffusion for 1 minutes in the slice of control, EAM or SB203580‐treated EAM heart. H and I, Quantification of dye diffusion measured as the fluorescence intensity vs distance from the edge (H) and as the area under the curve (I). Bar = 100 μm. Dotted line: the edge of dye transferred. White arrows: cutting edge at the apex of the heart

Based on the above results that the elevation of p^S368^Cx43 via p38 MAPK could cause the prolongation of QRS duration, it can be inferred that inhibition of the p38 MAPK should prevent the EAM‐induced prolongation of QRS duration. Inflammation is a complex process involving numerous mediators of cellular and plasma origin with elaborate, interrelated biological effects. Therefore, to avoid action of inflammation as many as possible, we applied p38 MAPK inhibitor SB203580 every 2 days after the most severe inflammation period (day 14) to day 20 of EAM, during which the QRS prolongation has been established. Figure 7A shows representative time courses of ECG recorded from the same rat of control, EAM or SB203580 treated EAM rats. In the control rats, the QRS duration (~18 ms) and QRS amplitude (~800 μV) were almost not altered from day 14 to day 21 (Figure 7B and C). In the EAM rats, the QRS duration was 21.5 ± 0.9 ms, 21.0 ± 0.3 ms and 21.0 ± 0.6 ms; while the QRS amplitude was 485 ± 62 μV, 435 ± 70 μV and 462 ± 58 μV on day 14, 17 and 21 of EAM respectively (Figure 7B and C). Treatment with SB203580 significantly reduced the EAM‐induced prolongation of QRS duration from 21.8 ± 1.1 ms on day 14 of EAM to 19.5 ± 0.5 ms and to 19.2 ± 0.5 ms on day 17 and day 21 of EAM respectively (Figure 7B). Meanwhile, the QRS amplitude was slightly increased from 392 ± 55 μV on day 14 to 619 ± 30 μV on day 21 of EAM (Figure 7C). In addition, SB203580 partially prevented the EAM‐induced elevation of the p^S368^Cx43 level (Figure 7D and E) and recovered the Cx43 distribution from the diffused pattern in EAM rats to the condensed manner in the intercalated disks as that in the control rats (Figure 7F). Furthermore, SB203580 partially restored the dye transfer ability of the heart, which was significantly impaired in the heart of EAM (Figure 7G‐I). These results suggest that inhibition of p38 MAPK activity could prevent not only the myocarditis induced elevation of p^S368^Cx43 but also the impairment of cell‐to‐cell communication and the prolongation of the QRS duration.

## 4 DISCUSSION

In this study, we provided several lines of evidence to support that the elevation of p^S368^Cx43 contributes to the prolongation of QRS duration in EAM. (i) The time course of changes in the QRS duration was coincided with that of the p^S368^Cx43 in EAM rats (Figures 1C and 2B); (ii) When the elevation of p^S368^Cx43 was suppressed by p38 MAPK blocker, the prolongation of QRS duration was prevented in EAM as well (Figure 7); (iii) When the level of p^S368^Cx43 was up‐regulated by IL‐1β, the QRS duration was prolonged even for the isolated normal heart (Figure 6).

In EAM rats, the QRS duration prolonged from day 10 to day 14, then reduced. Meanwhile, the p^S368^Cx43 level was also significantly increased, then decreased. What is the relationship between the prolongation of QRS duration and the elevation of the p^S368^Cx43 level? It has been demonstrated that mutation of Ser368 to Ala to prevent its phosphorylation makes the Cx43 form constitutively open channels,^33^ while mutation of Ser368 to Asp to mimic p^S368^Cx43 results in a closed state of the gap junctional channel,^39^ suggesting that up‐regulation of the p^S368^Cx43 could lead to impairment of cell‐to‐cell communication. As a matter of fact, many studies have shown that up‐regulation of p^S368^Cx43 reduces cell‐to‐cell communication.^30^, ^32^, ^40^ In this study, we demonstrated that the elevation of p^S368^Cx43 by the EAM serum or IL‐1β caused impairment of the cell‐to‐cell communication in the cultured cells. Extent of cell‐to‐cell communication through gap junction channels is one of the major factors to affect propagation of the electrical impulse through the heart.^19^ And impaired propagation of electrical impulse has been shown to be able to cause the prolongation of QRS duration.^21^, ^22^ Our study showed that blockade of p38 MAPK in EAM rats not only prevented the elevation of p^S368^Cx43 but also prevented the prolongation of QRS duration. Most importantly, in isolated hearts, which eliminates most of the factors that could influence ECG, perfusion of IL‐1β could still lead to the elevation of p^S368^Cx43, the impairment of cell‐to‐cell communication and thus the prolongation of QRS duration. Therefore, these results suggest that EAM induced elevation of p^S368^Cx43 might be responsible for the prolongation of QRS duration in EAM rats.

Many studies have shown that p^S368^Cx43 is up‐regulated through the PKC signalling pathway.^30^, ^32^, ^33^ In this study, we showed that p38 MAPK is involved in the up‐regulation of p^S368^Cx43 as well. Furthermore, we found that IL‐1β, one of the major inflammatory cytokines released in the early stage of EAM, could activate p38 MAPK, up‐regulate the p^S368^Cx43 level and suppress cell‐to‐cell communication. However, this does not exclude the contribution of PKC in the process. In fact, we found that blockade of either PKC or p38 MAPK could suppress the elevation of p^S368^Cx43. In good agreement with this result, it has been demonstrated that Cx43 forms a complex with protein kinases Src, p38 MAPK and PKC.^41^, ^42^ Therefore, it is highly likely that both PKC and p38 MAPK signalling pathways contribute to the elevation of the p^S368^Cx43 level in EAM.

Taken together, in this study, we identified a novel regulatory mechanism that up‐regulation of p^S368^Cx43 by IL‐1β via p38 MAPK signalling contributes to the prolongation of QRS duration in myocarditis. Thus, blockade of p38 MAPK in EAM rats prevents both the EAM‐induced elevation of p^S368^Cx43 level and the EAM‐induced prolongation of QRS duration, suggesting that p38 MAPK should be a novel therapeutic target for myocarditis.

## CONFLICT OF INTEREST

The authors declare no conflicts of interest.
