# Supplementary figures and images for: Up‐regulated Cx43 phosphorylation at Ser368 prolongs QRS duration in myocarditis
Source: J Cell Mol Med. 2018 Apr 17;22(7):3537–47. doi: 10.1111/jcmm.13631 (PMC6010859; doi:10.1111/jcmm.13631)

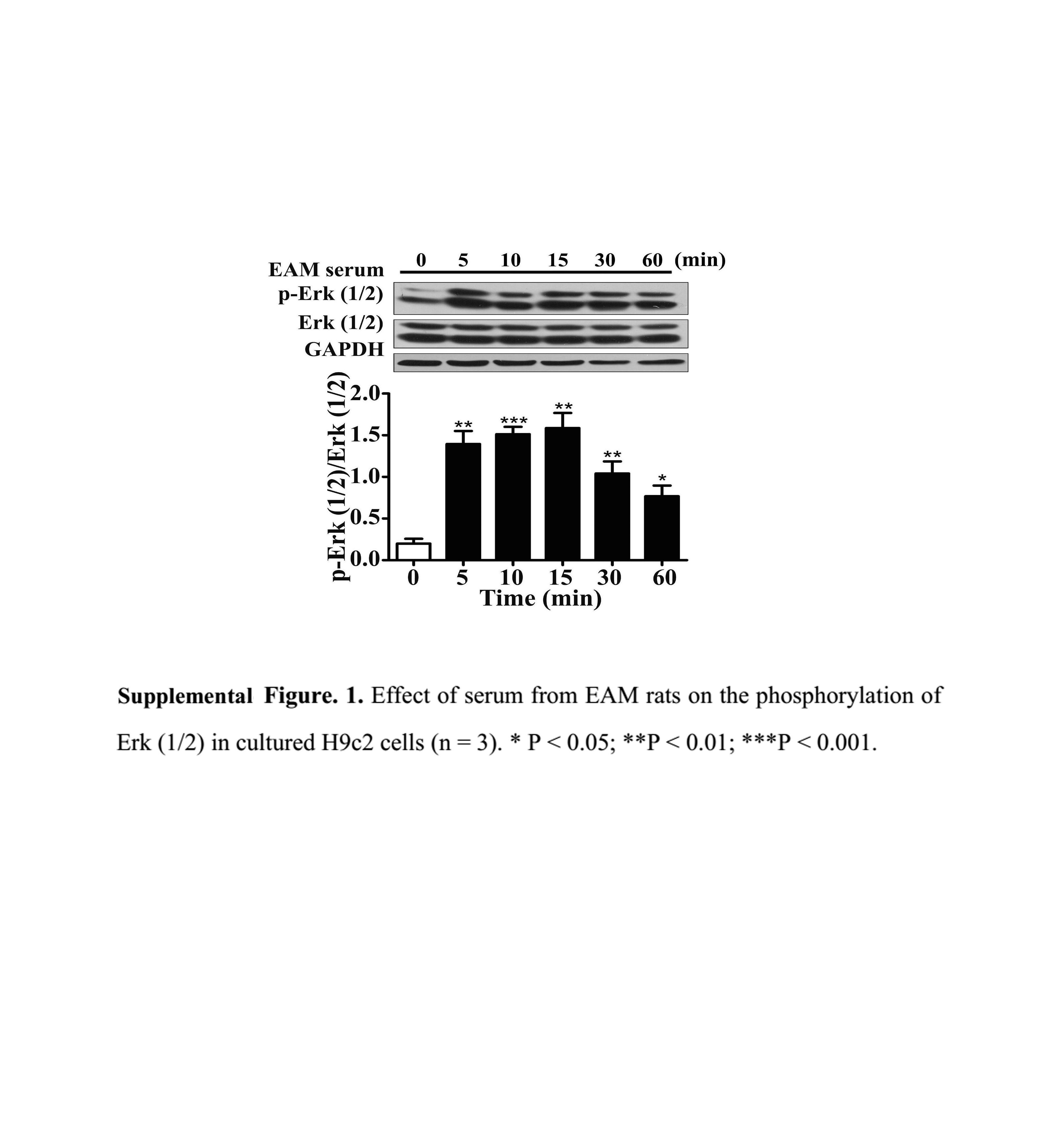

Supplement: Supplementary file 1 [file JCMM-22-3537-s001.jpg]

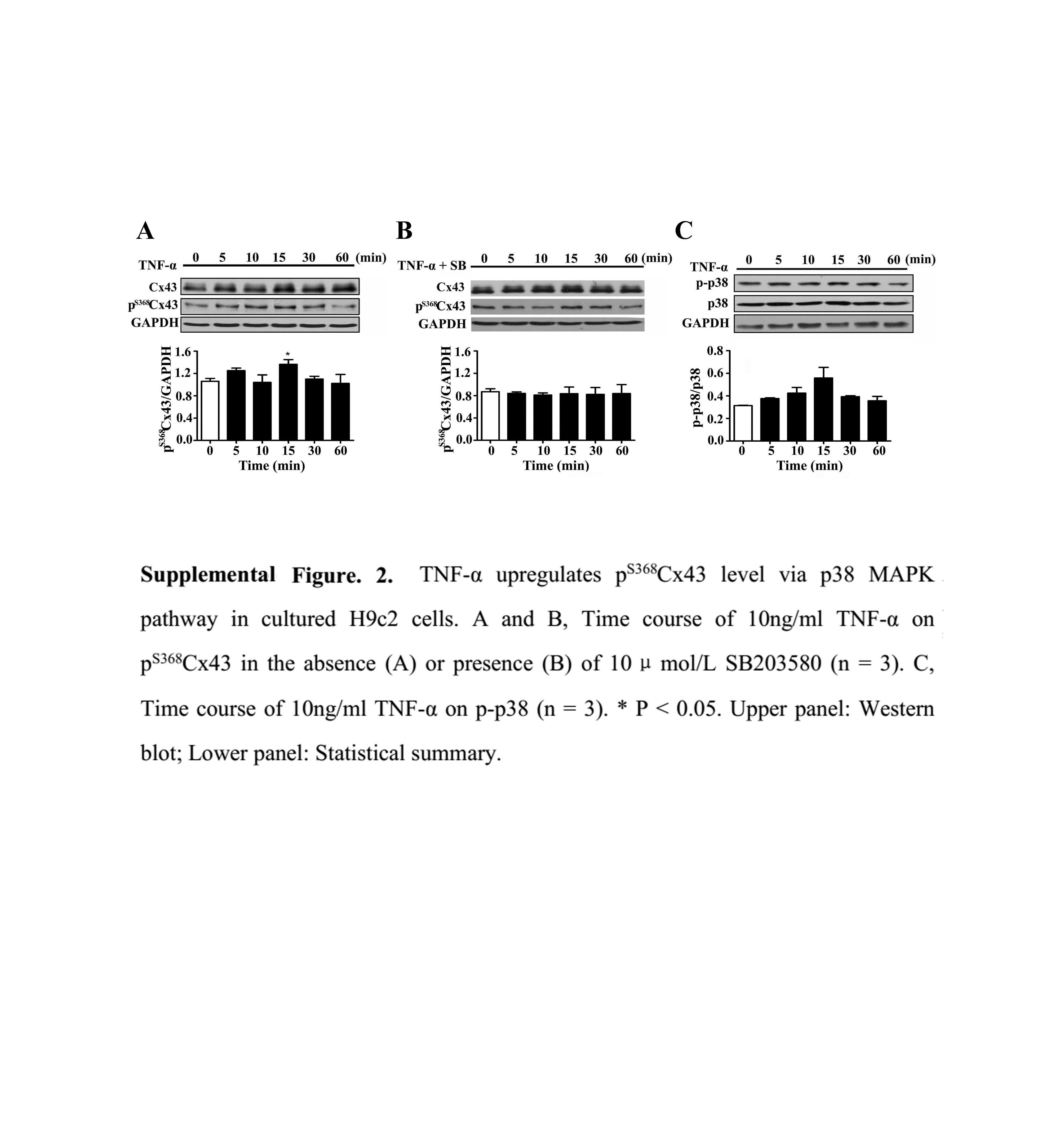

Supplement: Supplementary file 2 [file JCMM-22-3537-s002.jpg]
